# Supplementary material for: Open-source software library for real-time inertial measurement unit data-based inverse kinematics using OpenSim
Source: PeerJ. 2023 Apr 5;11:e15097. doi: 10.7717/peerj.15097 (PMC10082569; doi:10.7717/peerj.15097)
Supplement: Supplemental Information 1 [file peerj-11-15097-s001.pdf]

Supplemental Article S1

Article title: Open-source software library for real-time inertial measurement unit data-based inverse kinematics using OpenSim

Journal name: PeerJ

Authors: Jere Lavikainen, Paavo Vartiainen, Lauri Stenroth and Pasi A. Karjalainen

Corresponding author: Jere Lavikainen, jere.lavikainen@uef.fi, University of Eastern Finland, Department of Technical Physics

Placement of inertial measurement units on the subject

A total of 12 IMUs were placed on the subject's sternum, pelvis, upper arms, forearms, thighs, shanks, and feet as shown in the figure.

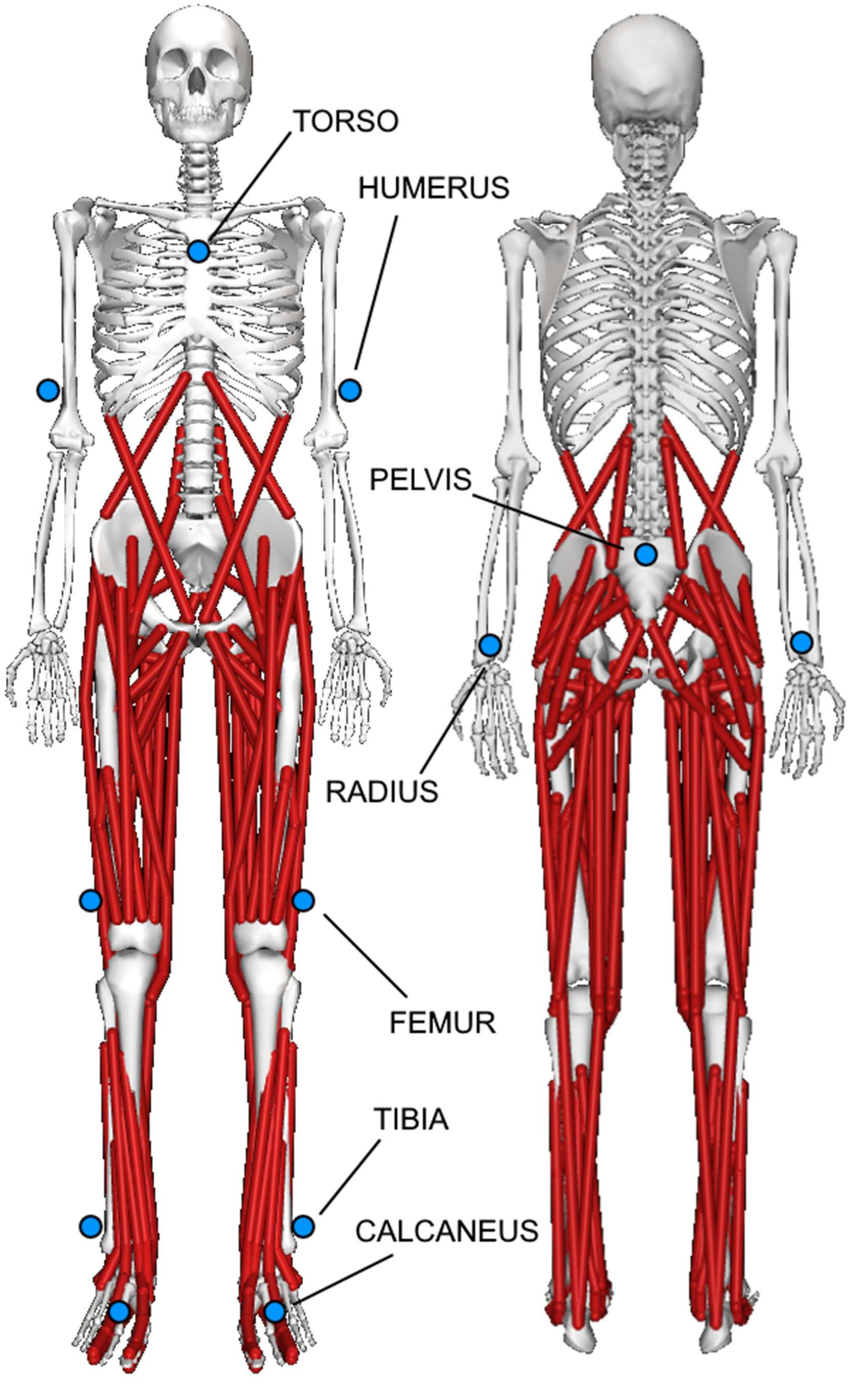
TORSO
HUMERUS
PELVIS
RADIUS
FEMUR
TIBIA
CALCANEUS

Velcro straps were used to fix the IMUs on the distal and lateral sides of the thighs, shanks, and upper arms, on the dorsal sides of the distal parts of the forearms, and behind the pelvis between the posterior superior iliac spines. One IMU was placed on the subject's sternum using a hook-and-loop fastener fold that was built into the subject's shirt. The foot IMUs were taped on the superior side of the metatarsi using athletic tape. The IMU placements were chosen based on prior experience in minimizing soft tissue error and matching orientation changes in the IMU to the orientation changes of the underlying bone.

Unlike in marker-based inverse kinematics, scaling the bony segments of the musculoskeletal model to the subject's dimensions is unnecessary in IMU-based inverse kinematics when no further analysis is done, and therefore, we did not scale the bony segments.

IMU calibration

During IMU calibration, all IMUs are associated with their corresponding body segments in the musculoskeletal model. At the instant of calibration, the orientations of the IMUs are assumed to represent the subject in the musculoskeletal model's default pose. In our measurements, we used a neutral standing position with arms hanging straight down and palms facing forward, i.e., the standard anatomical position.

The IMUPlacer class of OpenSim API was adapted to perform IMU calibration. First, orientation of the IMUs was transformed from the coordinate system of the IMUs to that of OpenSim. Second, heading correction was applied to adjust the orientations of the IMUs according to which way the subject was facing (as measured by the IMU on the pelvis). Prior to beginning calibration, the user had defined the transform from the IMU coordinate system to the OpenSim coordinate system and the IMU and its axis that corresponded to the forward-facing direction of the subject (i.e., the positive x-axis of the OpenSim model).

In the first phase, the user-given transform from the IMU coordinate system to the OpenSim coordinate system was applied on the IMU output quaternions. In the second phase, the pelvis IMU was used as the base IMU and the direction of the negative z-axis as the forward-facing direction. During the calibration procedure, the angle between the experimental IMU forward direction and the positive x-axis of the OpenSim model was calculated and all IMUs were rotated around the vertical axis of OpenSim by that angle, which ensured that IMU orientation readings were now in the forward-facing direction of the subject.

Our software library implemented this calibration procedure when the user pressed a key on the keyboard. The most recent orientations from the IMUs were then used to calibrate the model. Real-time IK could then be calculated using the calibrated model.

IK algorithm

The IK algorithm finds a set of generalized coordinates (i.e., joint angles and the global position and orientation of the model's pelvis or another segment) so that the weighted sum of squares of the orientation error is minimized. The orientation is expressed using an axis-angle representation as the angle component of the representation. Therefore, the orientation error is the difference between the angle component of experimental IMU orientation and the orientation of the corresponding body in the musculoskeletal model. In equation form, the term to be minimized is

$$\Sigma_i^N w_i x_i^2,$$

where N is the number of IMUs, w_i is the weight given to the orientation of the i'th IMU, and x_i is the angle component of the axis-angle orientation error of the i'th IMU (How IMU Inverse Kinematics Works, 2022). We used equal weights for all coordinates.

How IMU Inverse Kinematics Works. (2022, 9 26). Retrieved from OpenSim Documentation: https://simtk-confluence.stanford.edu:8443/display/OpenSim/How+IMU+Inverse+Kinematics+Works
